# Antibacterial, Antioxidant, and Anticholinesterase Activities of Plant Seed Extracts from Brazilian Semiarid Region

**DOI:** 10.1155/2013/510736

**Published:** 2013-12-10

**Authors:** Davi Felipe Farias, Terezinha Maria Souza, Martônio Ponte Viana, Bruno Marques Soares, Arcelina Pacheco Cunha, Ilka Maria Vasconcelos, Nágila Maria Pontes Silva Ricardo, Paulo Michel Pinheiro Ferreira, Vânia Maria Maciel Melo, Ana Fontenele Urano Carvalho

**Affiliations:** ^1^Departamento de Bioquímica e Biologia Molecular, Universidade Federal do Ceará, 60440-970 Fortaleza, CE, Brazil; ^2^Netherlands Toxicogenomics Centre, Maastricht University, Universiteitssingel 50, 6229ER Maastricht, The Netherlands; ^3^Departamento de Fisiologia e Farmacologia, Faculdade de Medicina, Universidade Federal do Ceará, 60430-270 Fortaleza, CE, Brazil; ^4^Departamento de Química Orgânica e Inorgânica, Universidade Federal do Ceará, 60440-970 Fortaleza, CE, Brazil; ^5^Departamento de Ciências Biológicas, Campus Senador Helvídio Nunes de Barros, Universidade Federal do Piauí, 64600-000 Picos, PI, Brazil; ^6^Programa de Pós-Graduação em Ciências Farmacêuticas, Universidade Federal do Piauí, Avenida Universitária, s/n, 64049-550 Teresina, PI, Brazil; ^7^Departamento de Biologia, Universidade Federal do Ceará, Campus do Pici, 60451-970 Fortaleza, CE, Brazil

## Abstract

The antimicrobial, antioxidant, and anticholinesterase activities of ethanolic seed extracts of twenty-one plant species from Brazilian semiarid region were investigated. The extracts were tested for antimicrobial activity against six bacteria strains and three yeasts. Six extracts presented activity against the Gram (−) organism *Salmonella choleraesuis* and the Gram (+) organisms *Staphylococcus aureus* and *Bacillus subtilis*. The MIC values ranged from 4.96 to 37.32 mg/mL. The *Triplaris gardneriana* extract presented activity against the three species, with MIC values 18.8, 13.76, and 11.15 mg/mL, respectively. Five extracts presented antioxidant activity, with EC_50_ values ranging from 69.73 **μ**g/mL (*T. gardneriana*) to 487.51 **μ**g/mL (*Licania rigida*). For the anticholinesterase activity, eleven extracts were capable of inhibiting the enzyme activity. From those, *T. gardneriana*, *Parkia platycephala* and *Connarus detersus* presented the best activities, with inhibition values of 76.7, 71.5, and 91.9%, respectively. The extracts that presented antimicrobial activity were tested for hemolytic assay against human A, B, and O blood types and rabbit blood. From those, only the *Myracrodruon urundeuva* extract presented activity (about 20% of hemolysis at the lowest tested concentration, 1.9 *µ*g/mL). Infrared spectroscopy of six representative extracts attested the presence of tannins, polyphenols, and flavonoids, which was confirmed by a qualitative phytochemical assay.

## 1. Introduction

Plants are considered natural sources of new compounds of medical and biotechnological interest, since they synthesize a large variety of bioactive compounds. An analysis into the sources of new drugs from 1981 to 2007 reveals that almost half of the drugs approved since 1994 were based on natural products. Among these, there are various examples of development of new drugs from plant sources [1]. Many researches have dedicated great effort to detect secondary metabolites with biological activities against cancer, microorganisms, tropical diseases, and others [2, 3]. Well-known examples of these compounds include flavonoids, phenols and phenolic glycosides, unsaturated lactones, sulphur compounds, saponins, cyanogenic glycosides, and glucosinolates [4–6].

In this context, the Brazilian semiarid region is a rich and underexploited source of bioactive molecules. This area is covered mainly by xeric shrublands known as “Caatinga.” This dry land vegetation grows over an area of 800,000 km^2^ in Northeastern Brazil, and out of 1,000 vascular plant species described many are endemic [7, 8]. Many of these plants are used in folk medicine by local communities to treat several diseases, pointing out the potential to discover new sources of useful compounds for several applications [9, 10]. Recently, our team has reported for seed ethanolic extracts of Brazilian Northeastern plants promising results of insecticidal activity against *Aedes aegypti *[11] and antiproliferative effects to tumor cell lines [12]. In order to extend our knowledge on the Brazilian semiarid vegetation, this work aimed to evaluate the presence of antimicrobial, anticholinesterase, and antioxidant activities in ethanolic seed extracts of twenty-one plant species. The antimicrobial activity was performed by the agar diffusion assay using pathogenic bacteria and yeast; antioxidant activity by the DPPH radical scavenging and anticholinesterase activity were measured. In addition, a hemolytic activity assay was done for antibacterial samples to assess the effects on nontarget cells.

## 2. Materials and Methods

### 2.1. Plant Collection

Mature wild seeds of twenty-one plant species were harvested (at least 500 g of each species) during the dry period (from January 2005 to March 2008) in the semiarid region of the Ceará State, Northeastern Brazil. Plants were identified by taxonomist Dr. Edson de Paula Nunes and voucher specimens were deposited at Herbarium Prisco Bezerra (EAC) at the Federal University of Ceará (Fortaleza, Brazil). The studied species are depicted in Table 1, which shows harvest information, voucher numbers, common names, and the traditional medicinal use by local population.

### 2.2. Preparation of Ethanolic Seed Extracts

Seeds of freshly collected plant material were separated from other plant materials, air-dried, and ground in a laboratory mill to a moderately fine powder (particle size ≤ 0.5 mm). Powdered material (500 g) was submitted to extraction with 99% ethanol (1.5 L) at room temperature (25–27°C) for 3 days. The supernatant was removed and filtered through Whatman number 1 paper. After that, the initial powder sample was still submitted to two extractions, which resulted in a final volume of extract near 4.5 L. The ethanolic extracts resulting from three consecutive extractions were mixed together then concentrated under reduced pressure in rotary evaporator until complete elimination of solvent, and stored in a freezer at −20°C.

A stock solution containing 50,000 *μ*g·mL^−1^ of each crude extract was prepared by suspending 50,000 mg of extract in 5 mL of pure dimethylsulphoxide (DMSO, Aldrich, Milwaukee, WI, USA) and mixed by sonication (model RK-100, Bandelin, Berlin, Germany) for 20 min. The volume was adjusted to 1,000 mL with distilled water to provide assay solutions as required.

### 2.3. Microorganisms

Six bacterial strains were used: *Staphylococcus aureus* (ATCC 25923), *Bacillus subtilis* (ATCC 6633), *Enterobacter aerogenes* (ATCC 13048), *Salmonella choleraesuis* (ATCC 10708), *Klebsiella pneumoniae* (ATCC 10031), and *Pseudomonas aeruginosa* (ATCC 25619). These standards were obtained from the American Type Culture Collection (ATCC) (Manassas, VA, USA). The stock culture was maintained on Mueller Hinton agar (HiMedia Laboratories Pvt. Ltd., Mumbai, India) medium at 4°C. Three pathogenic yeasts were used: *Candida albicans*, *C. krusei,* and *C. tropicalis*. These fungal strains were obtained from the Microbial Ecology and Biotechnology Laboratory, Biology Department of the Federal University of Ceara, Brazil. The stock cultures were maintained on Sabouraud Dextrose agar (HiMedia Laboratories Pvt. Ltd., Mumbai, India) medium at 4°C.

### 2.4. Antibacterial and Antifungal Activity

The disc diffusion method was followed for antibacterial and antifungal susceptibility test as described previously [16], with some modifications. Petri dishes were prepared by pouring 20 mL of Mueller-Hinton agar for bacteria and Sabouraud Dextrose agar for fungi and allowed to solidify. Dishes were dried and 0.1 mL of standardized inoculum suspension (10^6^ CFU/mL for bacteria and absorbance of 0.600 at 450 nm for fungi) was poured and uniformly spread. The excess inoculum was drained and the inoculum was allowed to dry for 5 min. The paper filter discs (7 mm of diameter) containing 20 *μ*L of each ethanolic extract were then applied and the dishes were incubated at 37°C for 18 h for bacterial growth and at the same temperature for 48 h for fungal growth. Tetracycline (30 mg/disc) was used as positive control for the antibacterial assay. Nystatin solution (100,000 IU/mL, EMS, SP, Brazil) (20 *μ*L/disc) was used as positive control for the antifungal assay. The inhibition zone was measured from the edge of the disc to the inner margin of the surrounding pathogens. All determinations were run in triplicate.

### 2.5. Minimum Inhibitory Concentration (MIC)

Minimum inhibitory concentrations of the seed ethanolic extracts were tested as described previously by the twofold serial dilution method. The test extract was dissolved in 5% DMSO to obtain 50 mg·mL^−1^ stock solution. Stock solution was diluted with distilled water to achieve 45, 40, 35, 30, 25, 20, 15, 10, and 5 mg·mL^−1^ or as required. The wet paper discs with dilutions were placed over the dish and the cultures were incubated in BOD incubators at 37°C for 18 h (bacteria) and 37°C for 48 h (yeasts). The lowest concentration, which inhibits the visible growth of tested organism after macroscopic evaluation, was determined as MIC. All determinations were run in triplicate.

### 2.6. Antioxidant Activity

The capacity to scavenge the 2,2-diphenyl-1-picrylhydrazyl (DPPH) free radical was monitored [17]. The diluted crude extracts (0.1 mL) in different concentrations (5 to 1,000 *μ*g·mL^−1^) were mixed with 3.9 mL of methanolic solution containing DPPH radicals (6.5 × 10^−1^ mol·L^−1^). The reduction of the DPPH radical was measured by monitoring continuously the decrease of absorption at 515 nm. The percentage scavenging of the DPPH radical was calculated according to the following formula: % scavenging effect = 100 ×[(*A*_DPPH_ − As)/*A*_DPPH_], where As is the absorbance of the solution when the sample has been added at a particular level and *A*_DPPH_ is the absorbance of the DPPH solution. The percentage of the remaining DPPH was plotted against the sample/standard concentration to obtain the amount of antioxidant necessary to decrease the initial concentration of DPPH by 50% (EC_50_). Based on the parameter EC_50_, the result was expressed in terms of *μ*g of seed extract per mL of DPPH in the reaction medium. All determinations were run in triplicate.

### 2.7. Anticholinesterase Activity

The anticholinesterase activity of the ethanolic extracts was measured as described by [18], with slight modifications. Acetylcholinesterase activity was measured using a 96-well microplate reader based on Ellman's method [19]. In this method the enzyme hydrolyzes the substrate acetylthiocholine resulting in the production of thiocholine which reacts with 5,5′-dithiobis(2-nitrobenzoic acid) (DTNB) to produce 2-nitrobenzoate-5-mercaptothiocholine and 5-thio-2-nitrobenzoate which can be detected at 405 nm. In the 96-well plates, 25 *μ*L of 15 mM acetylthiocholine iodide (ATCI) in water, 125 *μ*L of 3 mM DTNB in 50 mM Tris-HCl buffer containing 0.1 M NaCl and 0.02 M MgCl_2_·6H_2_O, 50 *μ*L of 50 mM Tris-HCl buffer containing 0.1% BSA, and 25 *μ*L of plant extract sample (5 mg·mL^−1^ in MeOH diluted ten times with 50 mM Tris-HCl buffer to give a concentration of 0.5 mg·mL^−1^) were added and the absorbance was measured at 405 nm every 30 s for three times. Then 25 *μ*L of 0.22 U·mL^−1^ of acetylcholinesterase from electric eel was added and the absorbance was again read every 45 s for eight times. Any increase in absorbance due to the spontaneous hydrolysis of the substrate was corrected by subtracting the rate of the reaction before the addition of the enzyme from the rate of the enzyme reaction. The percentage of inhibition was calculated by comparison of sample rates to a blank.

### 2.8. Hemolytic Activity Assay

The test was performed in 1.5 mL microtubes following the method described by [20] with some modifications. Firstly, a twofold serial dilution of each seed ethanolic extract was prepared with 0.9% NaCl ranging from 1,000 to 1.9 *μ*g·mL^−1^ and reserved. Then, 100 *μ*L of a 1% red blood cells (A, B, and O human blood types or rabbit blood) suspension was added to a new microtube containing 900 *μ*L of each seed extract dilution, which was then taken to incubator at 37°C for 1 h. After that, the tubes were centrifuged at 3,000 ×g for 5 min. The supernatant (200 *μ*L) was placed in a 96-well plate and led to a microplate reader (Epoch, BioTek, Vermont, USA) to measure the absorbance at 540 nm. The cell suspensions of each human blood type or those of rabbits (100 *μ*L) were mixed with distilled water or 0.9% NaCl (900 *μ*L) to obtain the absorbances for 100 and 0% of cell lysis, respectively. The percentage of hemolysis was calculated as follows: % hemolysis = Abs_test_/Abs_*pc*_× 100, where Abs_test_ = Abs_540_ of the 1% cell suspension treated with sample test and Abs_*pc*_ = Abs_540_ of the 1% cell suspension treated with distilled water. To calculate the relation between percentage of hemolysis and seed extract concentration, the hemolytic activity was expressed as the lowest seed extract concentration (*μ*g·mL^−1^) capable of causing hemolysis ≥ 20%. All determinations were run in triplicate.

### 2.9. Infrared Spectroscopy

The infrared spectrum was recorded from KBr discs on a FTIR Perkin Elmer model 16 PC spectrophotometer (PerkinElmer, Waltham, USA). Absorption maxima (*Ȟ*max) were reported in wavenumbers (cm^−1^). The spectra were used to determine organic groups indicative of the main classes of secondary metabolites.

### 2.10. Phytochemical Study

The ethanolic seed extracts were screened for phytochemical compounds such as phenols and tannins (reaction with ferric chloride), leucoanthocyanidins (heat treatment followed by alkalinization and acidification of sample), flavonoids and xanthones (reaction of magnesium granules with hydrochloric acid), steroids and triterpenes (extraction with chloroform, acetic anhydride, and sulfuric acid), saponins (foam production after water solubilization and stirring), and alkaloids (precipitation with Hager, Mayer, and Dragendorff reagents) [21]. These analyses were based on visual observation of color modification or precipitate formation after addition of specific reagents.

## 3. Results and Discussion

The ethanolic seed extracts did not show activity against the Gram (−) organisms *E. aerogenes*, *K. pneumonia, *and *P. aeruginosa* nor against the yeasts *C. albicans*, *C. krusei, *and *C. tropicalis*. MIC values obtained against the Gram (+) organisms *S. aureus *and *B. subtilis*, and the Gram (−) organism *S. choleraesuis* are shown in Table 1. Among all samples tested and microorganisms used, the seed extract of *S. obtusifolia *presented the lowest MIC value (4.96 mg·mL^−1^) against the pathogenic bacteria *S. aureus*. However, the species *T. gardneriana *showed the broadest spectrum of activity, being active against *B. subtilis*, *S. choleraesuis, *and *S. aureus* with MIC values of 18.8, 13.76, and 11.15 mg·mL^−1^, respectively. Although there are a great number of studies reporting antimicrobial activity of plant parts like leaves, roots and stems, antimicrobial screenings using specifically seed extracts are very rare. The reason for that is not easy to explain, since seeds are admittedly a rich source of compounds involved in plant defense [22]. A good example is the seed extract of *Bixa orellana *that has been described to have antimicrobial activity against six strains of bacteria, among these are *B. subtilis, S. aureus *and *P. aeruginosa*, and against the yeast *C. albicans *[23]. Likewise, guarana (*Paullinia cupana*) seed extracts presented strong antimicrobial activity against a huge number of damaging fungi and bacteria [24]. As to the potency of the antibacterial activities observed, these were much lower than those described for *Syzygium jambolanum *seed extracts against the same bacterial strains used in this work (MIC values ranging from 0.06 to 0.25 mg·mL^−1^), even when compared to the best result observed as described above for *S. obtusifolia *(MIC 4.96 mg·mL^−1^). In addition, the *S. jambolanum *seed extracts were also effective against damaging-fungi strains. The low potency of the ethanolic seed extracts could be due to the chemical composition of the samples, with low concentration of antimicrobial substances. In fact, the results of phytochemical study revealed that all seed extracts with activity against bacteria showed in their composition classes of secondary metabolites widely known as efficient antimicrobial compounds (alkaloids, flavones, flavonoids, phenols, saponins, and tannins) [25]. A fractionation of these samples in a polarity gradient could improve their activities. Therefore, the absence or low activity of the seed extracts could be a problem of concentration.

The hemolytic activity assay was performed with the seed extracts that were active against the microorganisms used. Among six species tested, only *M. urundeuva *caused hemolysis in all blood types (1% erythrocytes suspension of A, B, and O human blood types and rabbit blood) employed, being the lowest concentration tested (1.9 *μ*g·mL^−1^) capable of causing more than 20% hemolysis. These results show that although the antimicrobial extracts are not very potent, they are more specific to bacterial cells, except for *M. urundeuva *seed extract. It is well reported that some classes of plant secondary metabolites are potent hemolytic compounds [26], such as phenols that were also detected for *M. urundeuva *seed extract in the phytochemical study.

The results for antioxidant capacity are also shown in Table 1. Only five seed extracts were able to capture free radicals in the concentrations used according to DPPH method; these were *Hymenaea courbaril *(EC_50_ 247.95 *μ*g·mL^−1^), *L. rigida *(487.51 *μ*g·mL^−1^)*, L. tomentosa *(216.72 *μ*g·mL^−1^), and *T. gardneriana *(69.73 *μ*g·mL^−1^). The *L. tomentosa *and *T. gardneriana *seed extracts were more potent than the positive control vitamin C (EC_50_ 260.27 *μ*g·mL^−1^) and the second one was just a bit less potent than quercetin (EC_50_ 55.52 *μ*g·mL^−1^), one of the most potent antioxidant molecules. A previous study [27] showed that out of 25 plant extracts (hexane, dichloromethane, and methanol), only 12 presented some radical scavenging activity, and the most potent activity showed an IC_50_ value of 111.99 *μ*g·mL^−1^. This promising result for *T. gardneriana *could be attributed to components such as tannins, flavones, flavonoids, and especially phenols [28, 29]. Further steps of fractionation and purification may show the molecule(s) responsible for this excellent activity.

Concerning the anticholinesterase activity (Table 1), among the 21 ethanolic extracts tested, 11 presented inhibition of the cholinesterase enzyme. *T. gardneriana*, *P. platycephala* and *C. detersus *extracts presented the strongest activity, with inhibition values of, respectively, 76.7, 71.5, and 91.9% at the final concentration of 500 *μ*g/mL. These values are significant when compared to those obtained in previous studies [18], which have shown inhibition values of 17 plant extracts ranging from 9 to 81% using a single concentration of 1 mg/mL. Several plant-derived drugs, such as galantamine and rivastigmine, are used to inhibit AChE and thus increase endogenous levels of acetylcholine to improve cholinergic transmission [30]. Many studies described alkaloids as the main compounds capable of inhibiting AChE enzyme [31], but recent studies have pointed out several new classes of secondary metabolites as potent inhibitors, such as flavonoids [32], flavones [33], and xanthones [34], as well as steroids, terpenoids, oils, and other phenolic compounds [35]. Thus, the phytochemical analysis data in the present study corroborate previous molecular findings, since polyphenols were detected in all extracts. Additionally, the extract of *T. gardneriana* showed the presence of chalcones and aurones. Therefore, further studies should be conducted to assess the potential of these extracts and their respective molecules to inhibit AChE enzyme and thus offer a potential to fight neurodegenerative diseases such as Alzheimer's.

**Figure 1:**
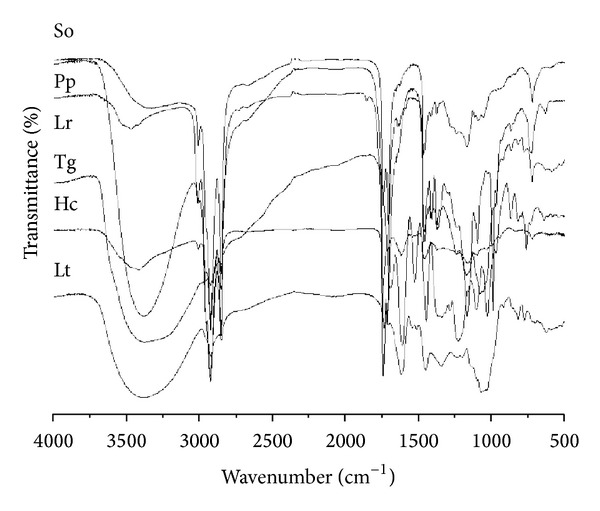
Infrared absorption spectra of some ethanolic extracts tested in this study. So: *Senna obtusifolia*; Pp: *Parkia platycephala*; Lr: *Licania rigida*; Tg: *Triplaris gardneriana*; Hc: *Hymenaea courbaril*; Lt: *Licania tomentosa*.

After running all the assays described above infrared spectroscopy with the most promising ethanolic seed extracts was also performed (*S. obtusifolia*, *P. platycephala*, *L. rigida*, *L. tomentosa,* and *T. gardneriana*) to confirm the results of phytochemical qualitative study. The phytochemical studies with these ethanolic extracts showed the presence of tannins and flavonoids, which were confirmed by infrared spectroscopy (Figure 1). The spectra obtained, between 1,800 and 800 cm^−1^, are present at the fingerprint zone of aromatic compounds, which can be found in tannins, polyphenols, and flavonoids [36]. Further steps of fractionation and purification may show the molecule(s) responsible for this excellent activity.

## 4. Conclusions

This study revealed antibacterial, antioxidant, and anticholinesterase activities in some seed ethanolic extracts of Brazilian semiarid region plants, emphasizing the antioxidant activity presented by *T. gardneriana *seeds. This showed a potent capacity to catch free radicals by DPPH assay. This promising result could be attributed to its composition featuring phenols, tannins, flavones and flavonoids. New phytochemical and molecular studies are underway to identify the bioactive compound(s) responsible for this activity.

## Figures and Tables

**Table 1:** Antibacterial (MIC), antioxidant, and hemolytic activities of ethanolic seed extracts of plants from Brazilian semiarid region^a^.

Family Botanical name Common name (locality: date of collection), Voucher number	Traditional medicinal use	Yield (%)^b^	Antioxidant activity (EC_50_ *µ*g/mL)^c^	Anticholinesterase activity (%)^d^	Antibacterial activity MIC (mg/mL)^e^		
					*S. Choleraesuis*	*B. subtilis*	*S. aureus*
*Anacardiaceae *							
*Myracrodruon urundeuva * Fr. All Aroeira-do-sertão (Araripe National Forest: 01/07) EAC 34865	Used to treat cutaneous and gynecological affections and kidney and respiratory problems; antiinflammatory, antiulcer, and healing [13]; antihistaminic and antimicrobial [14]; analgesic and antidiarrheal [15]	25.2	ND^f^	ND	ND	6.78	ND
*Schinopsis brasiliensis* Engler Braúna (Araripe National Forest: 01/07) EAC 35643	Used to treat nervousness and hysteria; analgesic [15]	2.88	ND	ND	37.32	ND	ND

*Caryocaraceae *							
*Caryocar coriaceum *Wittm. Pequi (Araripe National Forest: 09/05) EAC 35251	Not described	23.1	ND	63.4	ND	ND	ND

*Chrysobalanaceae *							
*Licania tomentosa* Benth Oiti (Coast zone: 01/08) EAC 40215	Not described	4.4	216.72	13.9	ND	ND	6.49
*Licania rigida* Benth Oiticica (Coast zone: 12/07) EAC 40216	Used to treat diabetes; anti-inflammatory [15]	28.1	487.51	52.4	ND	ND	8.63

*Connaraceae *							
*Connarus detersus* Planch Cabelo de negro (Araripe National Forest: 01/05) EAC 34733	Not described	28.5	ND	91.9	ND	ND	ND

*Fabaceae *							
*Adenanthera pavonina* L. Falso-sândalo (Coast zone: 08/07) EAC 38.697	Not described	6.12	ND	ND	ND	ND	ND
*Amburana cearensis* (Allemao) A.C. Smith Cumaru and amburana (Caatinga vegetation: 11/07) EAC 39618	Used to treat rheumatism, cold, and sinusitis; antispasmodic, healing, and anti-inflammatory [15]; analgesic [13]	6.43	ND	ND	ND	ND	ND
*Anadenanthera macrocarpa* (Benth.) Brenan Angico vermelho (Caatinga vegetation: 07/05) EAC 38389	Used to treat respiratory problems, gonorrhea, and diarrhea; astringent, antirheumatic, anti-inflammatory, sedative, and healing [15]	9.35	ND	54.1	ND	ND	ND
*Dioclea megacarpa* Rolfe Mucunã, olho-de-boi (Araripe National Forest: 01/05) EAC 38110	Not described	2.40	ND	ND	ND	ND	ND
*Enterolobium contortisiliquum* (Vell.) Morong Orelha-de-negro (Araripe National Forest: 09/05) EAC 38115	Not described	2.80	ND	ND	ND	ND	ND
*Hymenaea courbaril* L. Jatobá (Araripe National Forest: 01/05) EAC 38108	Not described	9.50	247.95	ND	ND	ND	ND
*Lonchocarpus sericeus* (Poiret) Kunth Ingá (Coast zone: 10/06) EAC 39615	Not described	10.60	ND	ND	ND	ND	ND
*Luetzelburgia auriculata* (Allemao) Ducke Pau-mocó (Caatinga vegetation: 11/06) EAC 40369	Not described	8.40	ND	ND	ND	ND	ND
*Parkia platycephala* Benth. Visgueiro (Araripe National Forest: 01/06) EAC38109	Not described	17.50	ND	71.5	ND	ND	ND
*Piptadenia moniliformis* Benth. Catanduva (Caatinga vegetation: 01/05) EAC35974	Not described	6.18	ND	50.2	ND	ND	ND
*Senna obtusifolia* (L.) H. S. Irwin & Barneby Mata pasto (Caatinga vegetation: 07/05) EAC39320	Not described	5.70	ND	ND	ND	4.96	ND

*Polygonaceae *							
*Triplaris gardneriana* Wedd. Pajeú (Caatinga vegetation: 11/06) EAC39600	Not described	26.0	69.73	79.8	18.8	13.76	11.15

*Rhamnaceae *							
*Ziziphus joazeiro* Mart Juá and juazeiro (Coast zone: 03/08) EAC 40366	Expectorant and antipyretic; used to treat skin, blood, stomach, and liver diseases, ulcer; antimicrobial [15]	7.22	ND	49.4	ND	ND	ND

*Sapindaceae *							
*Talisia esculenta *(A. St.-Hil) Radlk Pitomba (Coast zone: 03/08) EAC 40685	Not described	1.50	ND	61.24	ND	ND	ND
*Sapindus saponaria* L. Sabonete (Caatinga vegetation: 11/06) EAC39601	Not described	10.7	ND	58.60	ND	ND	ND

*Controls* ^ g^							
Quercetin	—	—	55.52	—	—	—	—
Vitamin C	—	—	260.27	—	—	—	—

^a^The values are means of a triplicate. The standard deviation was less than 5% of the mean.

^b^(Amount (g) of solid recovered from the ethanolic extract/amount (g) of seed flour used) × 100.

^c^Efficient concentration (EC), as *µ*g of sample required to decrease one g of the initial 2,2-diphenyl-1-picrylhydrazyl (DPPH^*·*^) concentration by 50%.

^d^Total inhibition of cholinesterase enzyme after comparison to the blank. Concentration tested: 500 *μ*g/mL.

^e^Minimum inhibitory concentration: the lowest concentration which did not show any growth of tested organism after macroscopic evaluation.

^f^ND: not detected.

^g^Substances that are frequently used as standard for antioxidant studies.
